# Complex hereditary peripheral neuropathies caused by novel variants in mitochondrial-related nuclear genes

**DOI:** 10.1007/s00415-022-11026-w

**Published:** 2022-03-02

**Authors:** Yu Hiramatsu, Yuji Okamoto, Akiko Yoshimura, Jun-Hui Yuan, Masahiro Ando, Yujiro Higuchi, Akihiro Hashiguchi, Eiji Matsuura, Fumihito Nozaki, Tomohiro Kumada, Kei Murayama, Mikiya Suzuki, Yuki Yamamoto, Naoko Matsui, Yoshimichi Miyazaki, Masamitsu Yamaguchi, Youji Suzuki, Jun Mitsui, Hiroyuki Ishiura, Masaki Tanaka, Shinichi Morishita, Ichizo Nishino, Shoji Tsuji, Hiroshi Takashima

**Affiliations:** 1 Department of Neurology and Geriatrics, Kagoshima University Graduate School of Medical and Dental Sciences, 8-35-1 Sakuragaoka, Kagoshima City, Kagoshima 890-8520 Japan; 2 Department of Physical Therapy, School of Health Sciences, Faculty of Medicine, Kagoshima University, Kagoshima, Japan; 3 Department of Pediatrics, Shiga Medical Center for Children, Shiga, Japan; 4 Kumada Kids Family Clinic, Shiga, Japan; 5 Department of Metabolism, Chiba Children’s Hospital, Chiba, Japan; 6 Department of Neurology, National Hospital Organization Higashisaitama Hospital, Saitama, Japan; 7 Department of Neurology, Tokushima University Graduate School of Medicine, Tokushima, Japan; 8 Department of Neurology, Hyogo Prefectural Awaji Medical Center, Hyogo, Japan; 9 Kansai Gakken Laboratory, Kankyo Eisei Yakuhin Co. Ltd., Seika-cho, Kyoto, Japan; 10 Department of Neurology, Yaizu City Hospital, Shizuoka, Japan; 11 Department of Neurology, Graduate School of Medicine, The University of Tokyo, Tokyo, Japan; 12 Institute of Medical Genomics, International University of Health and Welfare, Chiba, Japan; 13 Department of Computational Biology and Medical Sciences, Graduate School of Frontier Sciences, The University of Tokyo, Chiba, Japan; 14 Department of Neuromuscular Research, National Center of Neurology and Psychiatry (NCNP), National Institute of Neuroscience, Tokyo, Japan

**Keywords:** Peripheral neuropathy, Whole-exome sequencing, Nuclear genes, Mitochondrial disease

## Abstract

Mitochondrial disorders are a group of clinically and genetically heterogeneous multisystem disorders and peripheral neuropathy is frequently described in the context of mutations in mitochondrial-related nuclear genes. This study aimed to identify the causative mutations in mitochondrial-related nuclear genes in suspected hereditary peripheral neuropathy patients. We enrolled a large Japanese cohort of clinically suspected hereditary peripheral neuropathy patients who were mutation negative in the prescreening of the known Charcot–Marie–Tooth disease-causing genes. We performed whole-exome sequencing on 247 patients with autosomal recessive or sporadic inheritance for further analysis of 167 mitochondrial-related nuclear genes. We detected novel bi-allelic likely pathogenic/pathogenic variants in four patients, from four mitochondrial-related nuclear genes: pyruvate dehydrogenase beta-polypeptide (*PDHB*), mitochondrial poly(A) polymerase (*MTPAP*), hydroxyacyl-CoA dehydrogenase/3-ketoacyl-CoA thiolase/enoyl-CoA hydratase, beta subunit (*HADHB*), and succinate-CoA ligase ADP-forming beta subunit (*SUCLA2*). All these patients showed sensory and motor axonal polyneuropathy, combined with central nervous system or multisystem involvements. The pathological analysis of skeletal muscles revealed mild neurogenic changes without significant mitochondrial abnormalities. Targeted screening of mitochondria-related nuclear genes should be considered for patients with complex hereditary axonal polyneuropathy, accompanied by central nervous system dysfunctions, or with unexplainable multisystem disorders.

**Supplementary Information:**

The online version contains supplementary material available at 10.1007/s00415-022-11026-w.

## Introduction

Peripheral neuropathy has various causes, one of which is mitochondrial abnormalities. Mitochondrial-related nuclear genes, such as *MFN2* and *GDAP1* that are involved in mitochondrial dynamics, are major causes of Charcot–Marie–Tooth disease (CMT) [1, 2], the most common subtype of hereditary peripheral neuropathy. In *MFN2*, known as CMT2A and HMSN6A, phenotypes may cause optic atrophy, and in *GDAP1,* they may cause vocal cord paresis [2] so the spectrum of CMT has considerably broadened and multisystem involvement is frequently observed similar to other disorders caused by mutations in mitochondrial DNA (mtDNA) or mitochondria-related nuclear genes. Moreover, mitochondrial disorders associated with defects in mitochondrial DNA (mtDNA) maintenance and replication or defects in the respiratory chain complex are often associated with peripheral neuropathy [2]. Although the severity of these disorders is usually mild or subclinical, peripheral neuropathy can be severe and might be the main feature of a mitochondrial disorder [3]. Given that > 1100 mitochondrial-related nuclear genes have been identified and > 240 nuclear genes cause mitochondrial disorders [4, 5]; we speculate that more mutations in mitochondrial-related nuclear genes cause patients to manifest the CMT-like phenotype. In this study, through whole-exome sequencing (WES) data, we examined a large cohort of Japanese patients with clinically suspected hereditary peripheral neuropathy patients to determine the presence of variants in a panel of mitochondrial-related nuclear genes.

## Materials and methods

### Patient selection and extraction of genomic DNA

After preliminary exclusion of the *PMP22* duplication or deletion mutation, using fluorescence in situ hybridization or multiplex ligation probe amplification, we enrolled a nationwide cohort of 854 Japanese patients clinically suspected with pure or complex hereditary peripheral neuropathy, between April 2007 and July 2014. All of their clinical information, electrophysiological and radiological records, and pathological findings, were provided by local neurologists or pediatricians. The protocol was reviewed and approved by the Institutional Review Board of Kagoshima University (Kagoshima, Japan). All patients and family members provided written informed consent to participate in this study. The study conforms with the World Medical Association Declaration of Helsinki published on the website of the Journal of American Medical Association.

Genomic DNA was isolated from peripheral blood leukocytes, using the Qiagen Puregene Core Kit C (Qiagen, Valencia, CA, USA), or from the saliva, using the Oragene DNA self-collection kit (DNA Genotek, Ottawa, ON, Canada), according to the manufacturer’s protocol.

### Gene panel screening and WES

All 854 DNA samples were processed on 1 of the 2 types of CMT-related gene panel screening platforms. Between April 2007 and April 2012, 417 cases were screened using a customized MyGeneChip® CustomSeq® Custom Resequencing Array (Affymetrix, Inc., Santa Clara, CA, USA), targeting 28 genes known to cause CMT or related diseases following a protocol described previously [6]. Between May 2012 and July 2014, we used the Illumina MiSeq next-generation sequencing platform to screen 437 patients for 40 known CMT disease-causing and 20 candidate genes [7]. After target resequencing, we used WES to further analyze 399 mutation-negative patients, including 247 patients with autosomal recessive (AR) or sporadic inheritance.

We used a SureSelect v4 + UTRs or v5 + UTRs kit, then sequenced on Illumina HiSeq 2000® (Illumina, San Diego, CA, USA). WES data were aligned to the human reference genome (NCBI37/hg19) with Burrows–Wheeler Aligner [8], and variant call was performed using SAM tool [9], The CLC Genomic Workbench software program (Qiagen, Hilden, Germany) and an in-house R script were applied for variant annotation and filtering.

### Variant identification and segregation analysis

We concentrated on the WES variants in a list of 167 known mitochondrial-related nuclear genes, which was modified from the Baylor Genetics (https://www.bcm.edu/research/medical-genetics-labs/) BCM-MitomeNGS panel (Supplementary Table 1). Sanger sequencing was applied to validate the suspected pathogenic variants. We carried out segregation studies for other family members whenever available. All variants were checked against the single nucleotide polymorphism database (dbSNP: http://www.ncbi.nlm.nih.gov/SNP/), the gnomAD browser (https://gnomad.broadinstitute.org) as a global control database, the Human Genetic Variation Database (http://www.hgvd.genome.med.kyoto-u.ac.jp) and Japanese Multi Omics Reference Panel (jMorp 8.5 K: https://jmorp.megabank.tohoku.ac.jp/ijgvd/) as Japanese databases, and the in-house database to assess whether they were normal variants. Moreover, to perform in silico analysis, we used four prediction algorithms: PolyPhen-2 (http://genetics.bwh.harvard.educut/pph2, cut-off > 0.9), SIFT (http://sift.jcvi.org, cut-off < 0.05), PROVEAN (http://provean.jcvi.org/index.php, cut-off <  − 2.5), Mutation Taster (http://mutationtaster.org, scores ranging between 0 and 215, variants suspected of pathogenicity are classified as “disease-causing” and variants suspected of less pathogenicity are classified as “polymorphisms”). We interpreted variants according to the American College of Medical Genetics and Genomics and the Association for Molecular Pathology (ACMG/AMP) standards and guidelines [10].

### RNA extraction and reverse-transcription polymerase chain reaction (RT-PCR)

Total RNA was extracted from whole blood of Patient 4 using the PAXgene Blood RNA Kit (Qiagen). Subsequently, complementary DNA (cDNA) was produced with a high-capacity cDNA reverse-transcription kit (Applied Biosystems, Carlsbad, CA, USA) according to the manufacturer’s instructions. To analyze the effect of the splice site variant in the succinate-CoA ligase ADP-forming beta subunit (*SUCLA2*) gene, we amplified *SUCLA2* cDNA using the following primer pairs:

- Forward primer located in exon 4: 5′-GGAAGTTCACATGGTGGTGTC-3′
- Reverse primer located in exon 7: 5′-TGAGATTTGCCTTAGCAGCA-3′

### Clinical studies

Clinical findings and laboratory data, nerve conduction studies (NCS), and image examinations were based on the currently available information for all patients. The primary physician performed histological investigations of the sural nerve biopsy in Patient 1 as well as skeletal muscle biopsies in Patients 3 and 4, after obtaining informed consent. In Patient 4, respiratory chain enzyme activities in the skeletal muscle homogenate were also assayed as described earlier [11].

## Results

**Fig. 1:**
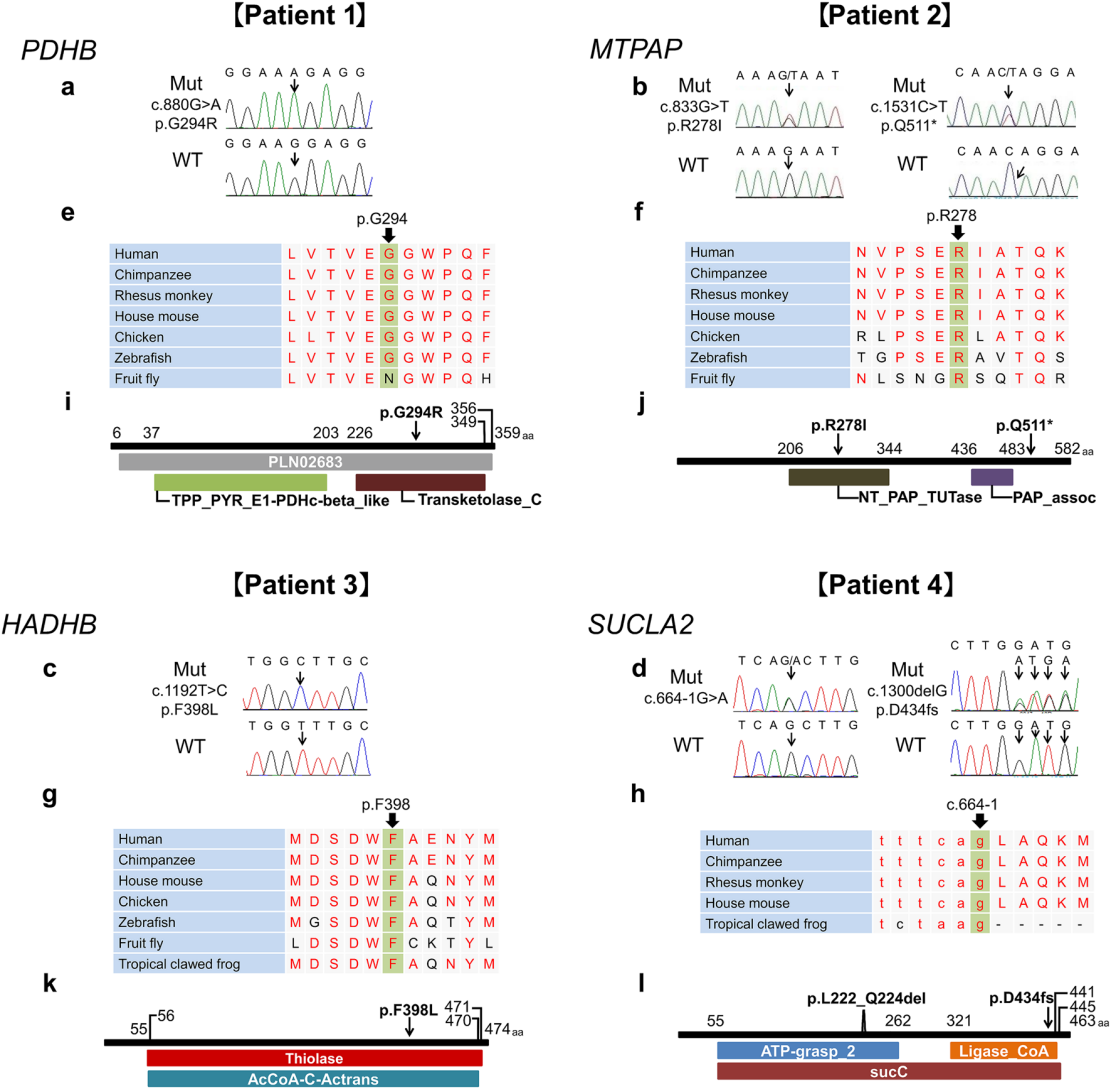
Localization and conservation of mitochondrial-related nuclear gene variants. **a**–**d** Sequencing chromatograms of c.880G > A (p.G294R) mutation in *PDHB* gene (**a**), c.833G > T (p.R278I) and c.1531C > T (p.Q511*) in *MTPAP* gene (**b**), c.1192 T > C (p.F398L) in *HADHB* gene (**c**), and c.664-1G > A and c.1300delG (p.D434Mfs*8) in *SUCLA2* gene (**d**). The patient number is noted above. Arrows indicate mutation sites. **e**–**h** Conservation analysis of amino acid sequences on the mutation sites in *PDHB* (**e**), *MTPAP* (**f**), and *HADHB* (**g**) genes and canonical GT-AG nucleotides (c.664-1 g) of the splice acceptor junctions in the *SUCLA2* gene (**h**). These mutation sites were highly conserved. **i**–**l** Predicted positions of amino acids affected and domain structure of PDHB protein (**i**), MTPAP protein (**j**), HADHB protein (**k**), and SUCLA2 protein (**l**) based on an NCBI conserved domain search (http://www.ncbi.nlm.nih.gov/Structure/cdd/wrpsb.cgi). Arrows indicate mutation sites. *aa* amino acids, *Mut* mutant, *WT* wild type

From WES data of 247 CMT patients with AR or sporadic inheritance, we concentrated all uncommon variants (allele frequency < 0.05) in the mitochondria-related 167 nuclear gene panel. Therein, bi-allelic variants were identified in four patients, from four distinct genes. These genes were pyruvate dehydrogenase, beta-polypeptide (*PDHB*), mitochondrial poly(A) polymerase (*MTPAP*), hydroxyacyl-CoA dehydrogenase/3-ketoacyl-CoA thiolase/enoyl-CoA hydratase, beta subunit (*HADHB*), and *SUCLA2*. These variants comprise c.880G > A (p.G294R) homozygous variants in *PDHB* (Fig. 1a), c.833G > T (p.R278I) and c.1531C > T (p.Q511*) compound heterozygous variants in *MTPAP* (Fig. 1b), c.1192 T > C (p.F398L) homozygous variants in *HADHB* (Fig. 1c), and c.664-1G > A and c.1,300delG (p.D434fs) compound heterozygous variants in *SUCLA2* (Fig. 1d). All the missense variants were novel and located at a highly conserved amino acid residues (Fig. 1e–h), and most variants were located in each protein domain region (Fig. 1i–l). All variants were absent in the control database and predicted to be damaging or deleterious during in silico analysis; the in silico analysis results as well as the classification by ACMG standards and guidelines are shown in Supplementary Table 2. The genetic and clinical presentations of the patients are summarized in Table 1.

**Table 1:** Genetic and clinical features of four patients with mitochondrial-related nuclear gene variants

Patient No	Patient 1		Patient 2		Patient 3		Patient 4	
Gene symbol	*PDHB*		*MTPAP*		*HADHB*		*SUCLA2*	
Mutation	c.880G > A (p.G294R)		c.833G > T (p.R278I)	c.1531C > T (p.Q511*)	c.1192 T > C (p.F398L)		c.1300delG (p.D434Mfs*8)	c.664-1G > A (p.L222_Q224del)
Genotype	Homo		C Hetero		Homo		C Hetero	
Age at the most recent exam (years)/Sex	37/F		37/M		56/M		4/F	
Onset	0 years		1 years		10 years		3 months	
Consanguinity	No		No		No		No	
Segregation	Yes		Yes		NA		Yes	
Initial symptom	Seizure		Gait disturbance		Languor of lower extremity		Hypotonia, Ptosis, Deafness, Scaphocephaly	
MMT^a^	2		1		3		3	
Sensory disturbance	Yes		Yes		Yes		No	
DTRs	All absent bilaterally		All absent bilaterally		All absent bilaterally		All absent bilaterally	
NCS								
dCMAP (mV)	Median 4.2	Tibial 2.53	Median 18.9	Tibial 1.98	Median 0.41	Tibial 1.49	Median 2.66	Tibial 0.96
MCV (m/s)	Median 46.4	Tibial 43.9	Median 59.7	Tibial 53.7	Median 54.5	Tibial 39.1	Median 61.6	Tibial 31.8
SNAP (μV)	Median 4.2	Sural 1.66	Median ND	Sural ND	Median 3.62	Sural ND	Median 29.1	Sural 8.4
SCV (m/s)	Median 53.8	Sural 45.6	Median ND	Sural ND	Median 48.4	Sural ND	Median 54.1	Sural 54.3
Other findings	Pes cavus Hallux valgus small-for-date infant Intellectual disability Strabismus VSD Ileus		Equinus foot Scoliosis Skin acne Vitamin B_12_ and folate deficiency		Pes cavus Hammer toes Brown urine Rhabdomyolysis		Encephalopathy Intellectual disability Dystonia Ophthalmoplegia Scoliosis Periodic vomiting Lactic acidosis Methylmalonic aciduria	

*C Hetero* compound heterozygous, *dCMAP* distal compound muscle action potential, *DTRs* deep tendon reflexes, *Homo* homozygous, *MCV* motor conduction velocity, *MMT* manual muscle testing, *NA* not available, *NCS* nerve conduction study, *ND* not detected (evoked), *SCV* sensory conduction velocity, *SNAP* sensory nerve action potential, *VSD* ventricular septal defect

^a^Scores indicating manual muscle testing (MMT) grade in the distal lower limbs

**Fig. 2:**
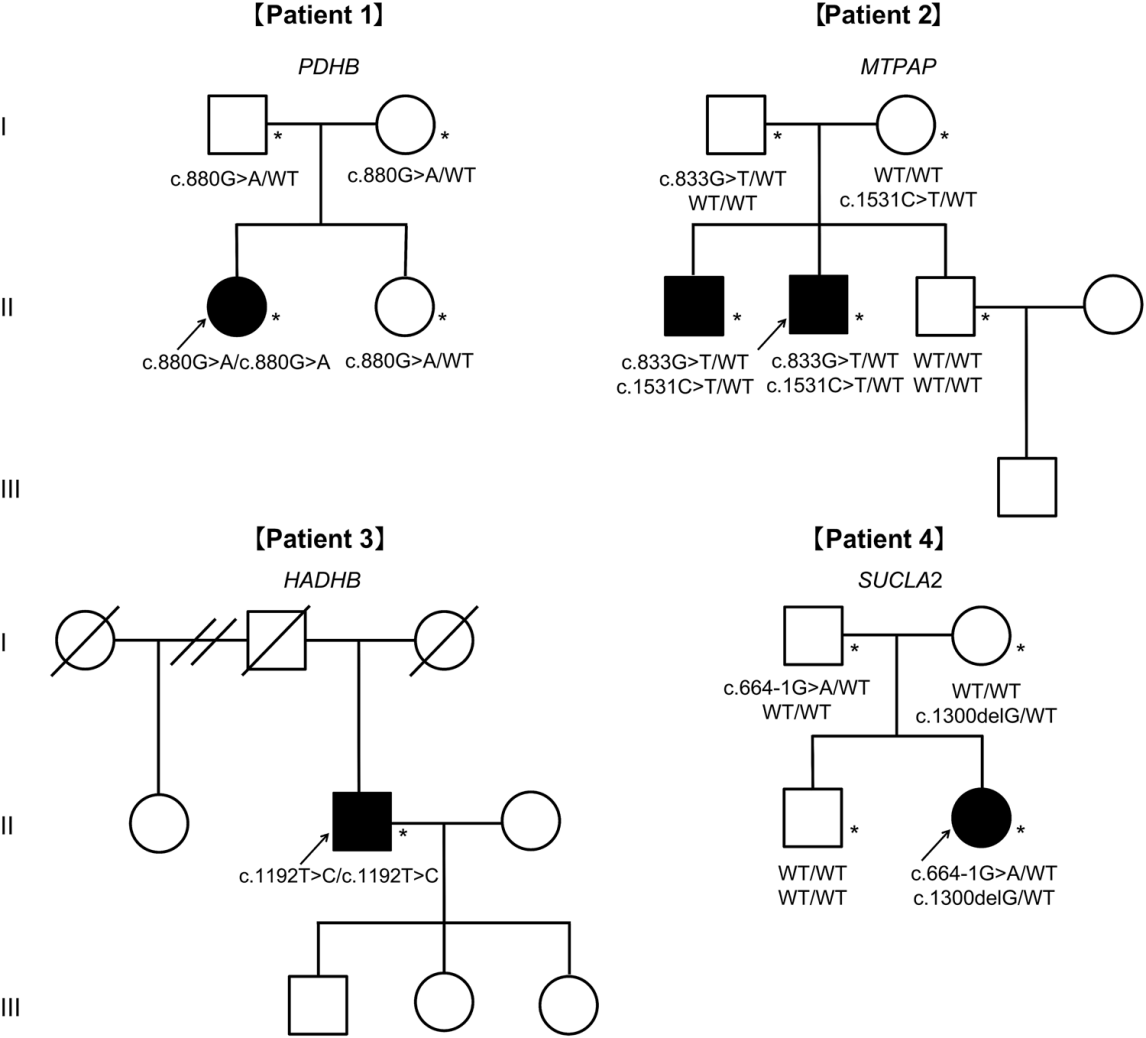
Pedigrees of families with novel mitochondrial-related nuclear gene variants. Genotypes of the variants are indicated at the bottom of the pedigree for each examined individual. Arrows and asterisks indicate the proband of each family and individual whose genome was applied for Sanger sequencing. *del* deletion, *WT* wild type

Segregation analyses were performed for the parents of all four patients, except Patient 3, whose parents were deceased. The same genotype was detected in an affected elder brother of Patient 2 (Fig. 2).

### Patient 1 [*PDHB* c.880G > A (p.G294R)]

**Fig. 3:**
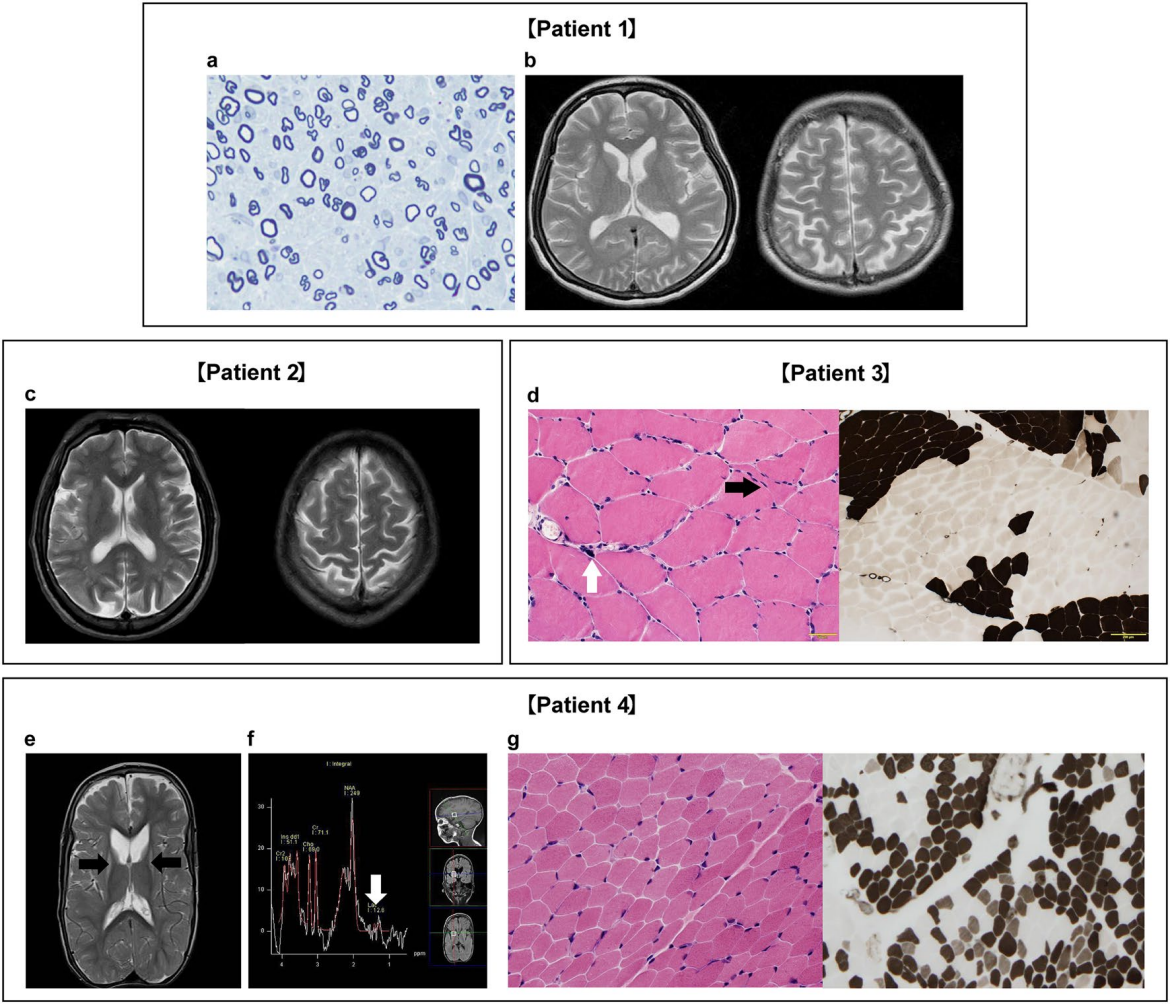
Brain imaging, peripheral nerve analysis, and analysis of skeletal muscle. **a** Toluidine blue staining of a sural nerve in Patient 1. Densities of large myelinated fibers are decreased, and fibers with thin myelin are occasionally noted. **b** Axial T2 images showing cerebral atrophy but no white matter lesions in Patient 1. **c** Axial T2 images showing cerebral atrophy, patchy areas, and white matter lesions in the temporal and occipital lobes in Patient 2. **d** Muscle biopsy showing mild variation in fiber size with a small angular fiber (black arrow) and pyknotic nuclear clump (white arrow) (hematoxylin and eosin staining) and marked fiber-type grouping (ATPase pH 10.6 staining) in Patient 3. **e** Axial T2 images showing scaphocephaly, cerebral atrophy, and hyperintensities in the bilateral putamen and caudate nuclei (black arrows) in Patient 4. **f** Pathological lactate accumulation was detected by magnetic resonance spectroscopy at short echo time (TE = 30 ms) in this area (white arrow). **g** Muscle biopsy showing mild variation in fiber size (hematoxylin and eosin staining) and fiber-type grouping (ATPase pH 10.4 staining) in Patient 4

This patient was a woman in her 30 s who was born weighing 2400 g. By 2 years of age, she had a ventricular septal defect and seizures four times. She began walking at 1 year and 6 months but had gait disturbance. The surgery of the strabismus was performed at age 4 and of the ileus at age 15. Gait disturbance progressed, and sural nerve biopsy was performed at age 25. A decrease in the density of large myelinated fibers with thin small myelinated clusters was revealed by nerve analysis (Fig. 3a). Simultaneously, adjustment disorder associated with mild-to-moderate intellectual disability was observed. Cerebral atrophy with no white matter lesions was shown by brain magnetic resonance imaging (MRI) (Fig. 3b). Clinically, the patient had weakness and atrophy of the distal limb muscles, pes cavus, and hallux valgus. Disturbance of the leg vibration sense and decreased tendon reflexes were also noted. Axonal polyneuropathy was revealed by NCS (Table 1).

### Patient 2 [*MTPAP* c.833G > T(p.R278I) c.1531C > T (p.Q511*)]

This male patient in his 30 s who had gait disturbance at the age of 1 year and 6 months. At 28 years, scoliosis, equinus foot, and paresthesia were present, and he could not walk. Sensory-dominant axonal polyneuropathy was revealed by NCS (Table 1), and he had skin acne through the lumbar region from the head. Low levels of vitamin B_12_ (70 ng/mL; normal: 180–914 ng/mL) and folate (1.3 ng/mL; normal: > 3 ng/mL) were revealed in laboratory investigations, but the intrinsic factor antibody and gastric parietal cell antibody were negative. An upper gastrointestinal endoscopy showed only atrophic gastritis. Patchy T2 hyperintensities in the white matter of the temporal and occipital lobes (Fig. 3c) were revealed by brain MRI.

During elementary school, his elder brother, who had the same *MTPAP* variants, also had gait disturbance, amblyopia, and scoliosis, and he began using a wheelchair at 38 years of age. We observed hypoesthesia and optic atrophy, and axonal polyneuropathy was revealed by NCS. His vitamin B_12_ levels were low, and despite supplementation, the symptoms worsened.

### Patient 3 [*HADHB* c.1192 T > C (p.F398L)]

Details about this case were previously established [12]. A man in his 50 s had languor of the lower extremity and brown urine after exercise at the age of 10. For a while, symptoms improved but recurred at age 45, and gait disturbance progressed. At age 55, deep tendon reflexes were absent, and muscle weakness and atrophy in his distal lower limbs were present. His senses of vibration and position were deeply disturbed, and sensory ataxia was present. Axonal sensorimotor polyneuropathy was shown by NCS (Table 1) and normal creatine kinase levels by laboratory investigations. After gene analysis, he suffered from recurrent rhabdomyolysis. Neurogenic changes, such as marked fiber-type grouping were revealed by open muscle biopsy (Fig. 3d), but there was no mitochondrial abnormality. His 3-hydroxy-tetradecanoyl carnitine (C_14_-OH) level was slightly increased (0.06 nmol/mL; normal: < 0.05 nmol/mL).

### Patient 4 [*SUCLA2* c.1300delG (p.D434Mfs*8) c.664-1G > A (p.L222_Q224del)]

Details about this case were previously established [13]. This infant girl was born weighing 2,450 g. At 3 months, ptosis, deafness, scaphocephaly, and axial hypotonia were observed. At 7 months, profound intellectual disability was noted. At 1 year, she developed dysphagia, and tube feeding was started. At 2 years, gastroesophageal reflux and scoliosis were observed accompanied by cyclic vomiting and hypercapnia during sleep. NCS showed that axonal polyneuropathy predominantly affected the motor nerves (Table 1). Laboratory tests showed increased levels of mild methylmalonic aciduria (34.1 μg/mg creatinine; normal: < 10 μg/mg creatinine), blood lactate (58.5 mg/dL; normal: 4–17 mg/dL), and pyruvate (2.17 mg/dL; normal: 0.3–0.9 mg/dL), and cerebrospinal fluid lactate and pyruvate levels were 27.9 mg/dL and 1.81 mg/dL, respectively. Brain MRI revealed cerebral atrophy and T2 hyperintensities in the bilateral putamen and caudate nuclei (Fig. 3e) and a lactate peak in magnetic resonance spectroscopy (Fig. 3f). Therefore, Leigh syndrome was suspected; however, there was no known mitochondrial DNA mutation in peripheral blood lymphocytes. Open muscle biopsy showed a neurogenic change in the fiber-type grouping (Fig. 3g), but no insufficient cytochrome *c* oxidase activity, ragged red fibers, or increased staining for succinate dehydrogenase were observed. Hence, she was tentatively diagnosed with CMT2 or other complex hereditary peripheral neuropathy.

After the gene analysis, the muscle respiratory chain enzyme activities showed deficiencies in complexes I (45%) and IV (43%), whereas complex II (the only complex that does not contain mtDNA-encoded proteins) was normal (105%; each activity was expressed as the citrate synthase ratio).

**Fig. 4:**
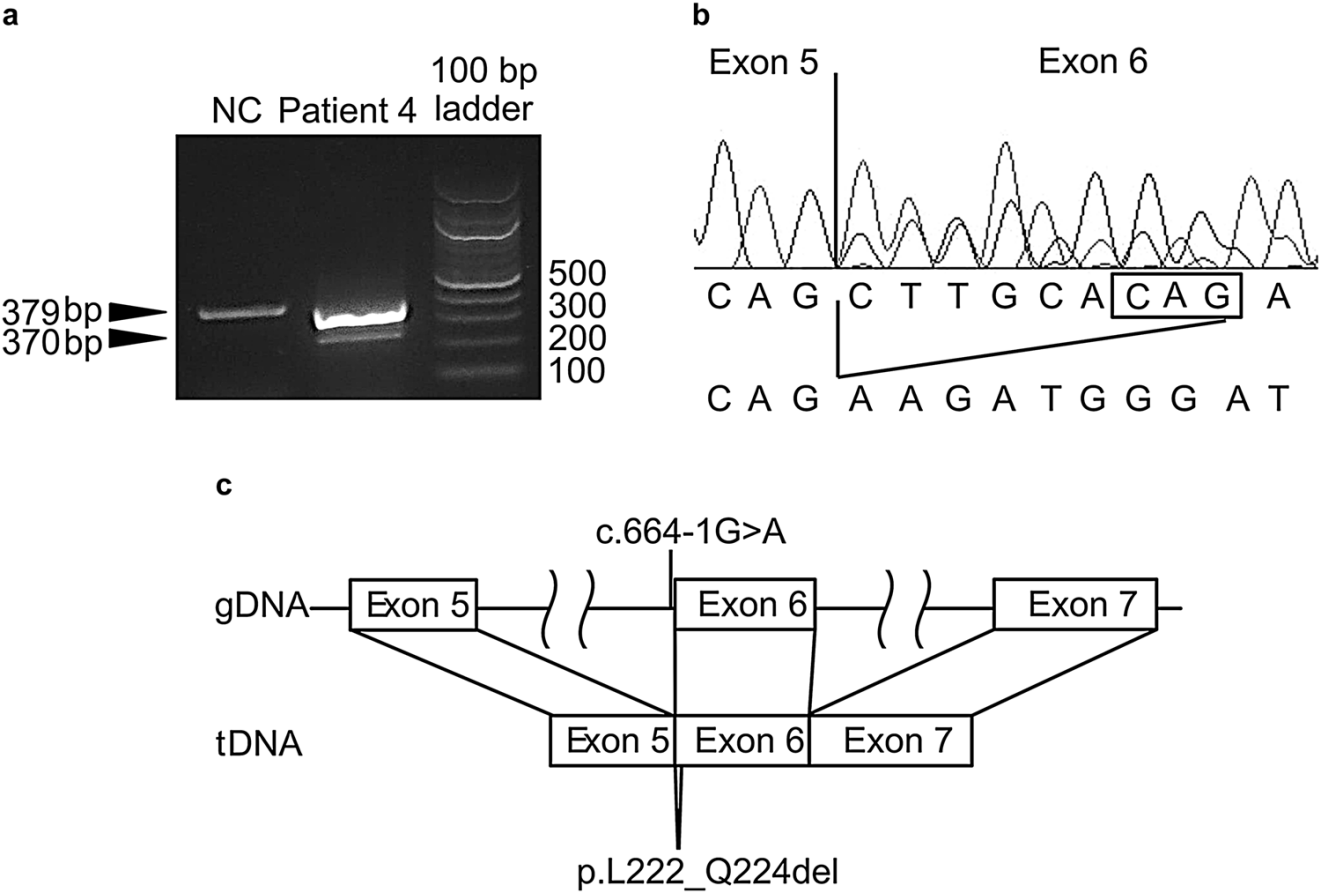
RNA analysis of *SUCLA2*. **a** Agarose gel electrophoresis of cDNA fragments obtained from the RT-PCR of Patient 4. Patient 4 had the expected 379 bp band and an additional 370 bp band, which is the result of the deletion of exon 6 in one allele. **b**, **c** Sequence chromatogram of the RT-PCR product from Patient 4 showing heterozygote 9 bp deletion in exon 6. *bp* base pairs, *NC* normal control

Regarding heterozygous splice acceptor site variant (c.664-1G > A) in intron 5, we detected two RT-PCR products comprising a wild-type band and a smaller band at 370 bp (Fig. 4a). Sanger sequencing of the RT-PCR product revealed 9 bp heterozygous deletion (Fig. 4b), leading to the in-frame deletion of three amino acids in exon 6 of *SUCLA2* (p.L222_Q224del; Fig. 4c).

## Discussion

Using WES, we performed further genetic analysis targeting 167 mitochondrial-related nuclear genes among a group of patients with clinically suspected with pure or complex hereditary peripheral neuropathy. In four patients, we identified four disease-causing genes, of which variants in *PDHB*, *MTPAP*, and *SUCLA2* have been reported not as a cause of CMT but of other diseases associated with symptomatic or subclinical peripheral neuropathy [14–16]. Although recessive mutations in *HADHB* have been assumed to be associated with axonal CMT, no pathophysiological mechanism was elucidated [17, 18]. We indicated the pathological significance of the *PDHB* and *HADHB* genes using *Drosophila* neuron-specific knockdown models in a previous study [19, 20]. Although all four patients presented with various phenotypes, motor and sensory axonal neuropathy was the major clinical feature.

The pyruvate dehydrogenase alpha (*PDHA*) and *PDHB* encode pyruvate dehydrogenase, a component enzyme of the pyruvate dehydrogenase multienzyme complex in mitochondria [21]. Recessive *PDHB* mutation causes pyruvate dehydrogenase complex (PDC) deficiencies with severe clinical consequences that primarily affect the nervous system, such as developmental delay, seizures, central hypotonia, ataxia, peripheral neuropathy, microcephaly, congenital brain malformations, and degenerative changes, such as Leigh syndrome [14, 21, 22]. Given that cases of thiamine-sensitive PDC deficiency have been reported and a ketogenic diet may alleviate the symptoms [21], it is important to diagnose this disease. In 3–21% of patients, peripheral neuropathy has been found to be linked to PDC deficiencies [21], whereas progressive peripheral neuropathy in adult patients has been linked only to mutations in *PDHA1* [22]. In Patient 1, in addition to peripheral neuropathy, central nervous system involvement was suggested by multiple manifestations, comprising seizures, intellectual disability, and cerebral atrophy in brain MRI. The G294R variant is located at the transketolase coding region of the C-terminal domain of the PDHB protein (Fig. 1i) and is close to the previously reported mutations of PDC deficiencies [14].

*MTPAP* gene encodes the mitochondrial polyadenylation polymerase, involved in DNA maintenance and repair [23]. Recessive mutation in *MTPAP* would result in delayed DNA repair, elevation of the level of DNA double-strand breaks, reactive oxygen species, and cell death after irradiation, leading to spastic ataxia and optic atrophy or encephalopathy. A previous case report described progressive lower motor neuron involvement due to *MTPAP* mutation [15] but progressive peripheral neuropathy, especially sensory nerves, without pyramidal signs, as observed in family 2, has not previously been described.

Importantly, low levels of vitamin B_12_ and folate, as found in Patient 2 and his brother, might also cause peripheral neuropathy. Folate and vitamin B_12_, especially methylcobalamin, are coenzymes for cytosolic methionine synthase involved in vital cellular processes, such as methylation and DNA synthesis [24, 25]. Bi-allelic mutations of *MTPAP* would elevate both DNA double-strand breaks and cell death, whereas over-consumption of vitamin B_12_ and folate for DNA synthesis could cause deficiencies of these substances. Despite the normalization of vitamin B_12_ and folate levels by supplementary treatment, their symptoms progressed, suggesting other contributive reasons. In family 2, segregation analysis confirmed that p.R278I and p.Q511*of *MTPAP* are compound heterozygotes, which would affect both DNA strands, thereby influencing the MTPAP protein function. The p.R279I variant is located in the nucleotidyltransferase (NT) domain of poly(A) polymerases and terminal uridylyl transferases (Fig. 1j); no mutation is reported in this domain. Because the p.Q511* variant is located in the last exon, and loss-of-function has not been a recognized mechanism in *MTPAP*-related disease, this variant was classified as “strong,” as per the ACMG standards and guidelines. Further investigations are needed to clarify the role of vitamin B_12_ and folate in the pathogenesis of *MTPAP*-related peripheral neuropathy.

The alpha and beta subunits of the mitochondrial trifunctional protein (MTP) are encoded by the *HADHA* and *HADHB* genes [26]. These subunits catalyze three steps in the beta-oxidation of fatty acids, including the long-chain 3-hydroxyacyl-CoA dehydrogenase step. Recessive *HADHB* mutation results in the dysfunction of the beta-oxidation of fatty acids, leading to MTP deficiency, characterized in a severe heterogeneous syndrome, such as cardiomyopathy, recurrent Leigh-like encephalopathy, hepatopathy, and neonatal or unexpected infant death [27]. In contrast, the milder form is characterized by later-onset progressive axonal peripheral neuropathy (approximately 50–80%) and myopathy with or without episodic myoglobinuria [17, 26]. Phenylalanine 398 is located in the thiolase, C-terminal domain of HADHB protein (Fig. 1k), and is close to known mutations linked to the milder form [26]. After the genetic diagnosis, Patient 3 experienced recurrent episodes of brown urine after exercise and rhabdomyolysis, and his C_14_-OH level increased slightly; all these characteristic phenotypes were considered supportive evidence to his diagnosis. As described for Patient 3, peripheral neuropathy may be the main symptom in patients with *HADHB* mutations, or at least in certain stages of the disease course.

The *SUCLA2* gene is involved in the citric acid cycle and mtDNA synthesis [28]. In the citric acid cycle, SUCLA2 catalyzes the reversible formation of succinate and ATP from succinyl-CoA and ADP. This protein also interacts with nucleoside diphosphate kinase, which is involved in mtDNA synthesis [28]. Recessive *SUCLA2* mutation causes mtDNA depletion syndrome, which could cause mild methylmalonic aciduria, recurrent vomiting, hypotonia, dehydration, respiratory distress, neonatal encephalopathy, and progressive lethargy [16]. Peripheral neuropathy could also be observed, as described for Patient 4. We identified two heterozygous variants in *SUCLA2* from Patient 4: frame shift (c.1300delG) and splice site (c.664-1G > A); the latter was found to produce a protein that is three amino acids smaller than normal (p.L222_Q224del). The c.1300delG variant, located close to the 3′-terminal of SUCLA2, is likely to produce a truncated protein (Fig. 1l). Taken together with the segregation analysis, we classified these bi-allelic variants as pathogenic/probably pathogenic.

**Fig. 5:**
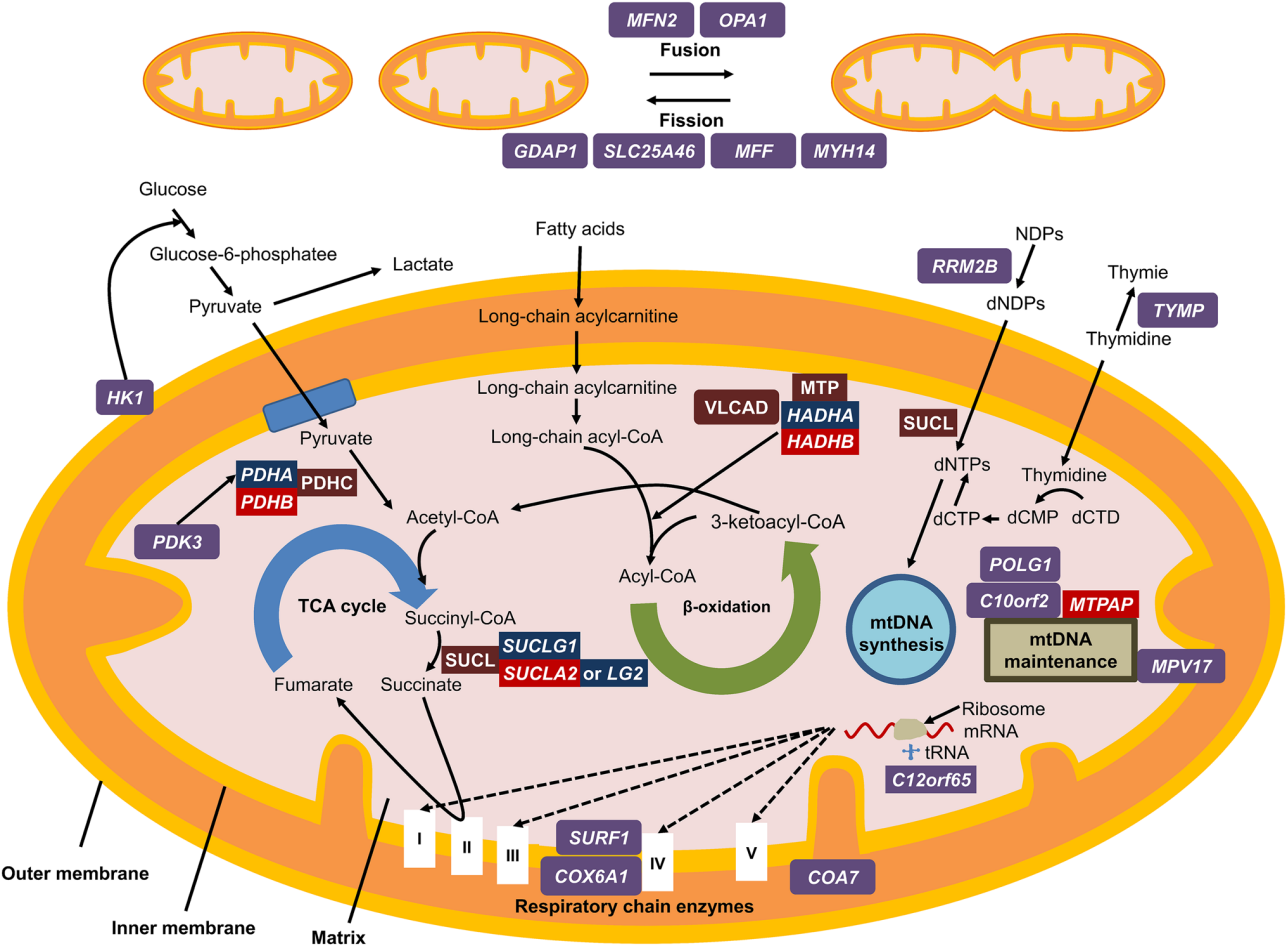
Overview of the mechanisms underlying the main mitochondrial-related genes causing peripheral neuropathy and genes described in this report. Genes edged in purple are the main mitochondrial-related genes and those edged in red are the genes described in this report. PDHC comprises two alpha subunits *(PDHA)* and two beta subunits *(PDHB)*. MTP comprises alpha subunits *(HADHA)* and beta subunits *(HADHB)*. SUCL comprises an alpha subunit encoded by *SUCLG1* and a beta subunit encoded by either *SUCLA2* or *SUCLG2*. *CoA* coenzyme A, *dCMP* deoxycytosine monophosphate, *dCTD* deoxycytidine, *dCTP* deoxycytosine triphosphate, *dNDPs* deoxynucleoside diphosphates, *mtDNA* mitochondrial DNA, *MTP* mitochondrial trifunctional protein, *NDPs* nucleoside diphosphates, *PDHC* pyruvate dehydrogenase complex, *SUCL* succinyl-CoA ligase, *TCA* tricarboxylic acid, *VLCAD* very-long-chain acyl-CoA dehydrogenase.

Peripheral nerves have long axons wrapped in myelin lamellae provided by Schwann cells and are highly dependent on energy metabolism, ATP synthesis might eventually be influenced by any abnormalities in energy production and depletion of mitochondria, leading to peripheral neuropathy. Moreover, various mitochondrial dysfunctions, such as dysfunctions in mitochondrial energy production or PDC, or assembling mitochondrial respiratory chain complex have been reported to cause CMT (Table 2) [2, 29–35]. Pathological mechanisms of each gene remain unclear, because peripheral nerves have long axons wrapped in myelin lamellae provided by Schwann cells and are highly dependent on energy metabolism; however, ATP synthesis might eventually be influenced by any abnormalities in energy production and depletion of mitochondria, leading to peripheral neuropathy (Fig. 5). As observed in patients with bi-allelic variants in *PDHB* and *SUCLA2*, in mitochondrial disorders, multisystem manifestation representative of central nervous system involvement is a common feature suggestive of clinical diagnosis. Conversely, like *MTPAP* and *HADHB*, these genes could hardly result in central nervous system abnormalities; thus, more attention should be paid to clinical assessments, particularly for certain symptoms that transiently emerged in their disease course and naturally disappeared with age. Moreover, because of the clinical diversity of these patients, peripheral neuropathy has always been recognized as part of the mitochondrial disorder rather than of CMT. Heterogeneous phenotypes of both mitochondrial disorders and CMT make the clinical diagnosis of either difficult. However, the development of diagnostic genetics has facilitated diagnoses made on a genetic basis.

**Table 2:** Genetic pathophysiology, phenotype, inheritance pattern, and neuropathy type of the mitochondrial-related nuclear genes described in this report (upper table) and previously reported genes (lower table)

Gene	Pathophysiology	Phenotype	Inheritance	Neuropathy type
*PDHB*	Pyruvate dehydrogenase complex	Pyruvate dehydrogenase E1-beta deficiency	AR	Sensory-motor axonal
*MTPAP*	mtDNA maintenance and repair	SPAX4 Cellular radiosensitivity	AR	Sensory-motor axonal
*HADHB*	Mitochondrial energy production (beta-oxidation)	Trifunctional protein deficiency	AR	Sensory-motor axonal
*SUCLA2*	Mitochondrial energy production (tricarboxylic acid cycle), mtDNA synthesis	Mitochondrial DNA depletion syndrome 5	AR	Sensory-motor axonal
*MFN2*	Mitochondrial dynamics (fusion)	CMT2A2, HMSN6 (CMT6A)	AR and AD	Sensory-motor axonal
*OPA1*	Mitochondrial dynamics (fusion)	Optic Atrophy I, Mitochondrial DNA depletion syndrome	AD	Sensory-motor axonal
*GDAP1*	Mitochondrial dynamics (fission)	CMT4A, CMT2K, CMTRIA, CMT with vocal cord paresis	AR and AD	Sensory-motor axonal (with or without secondary demyelinating changes)
*SLC25A46*	Mitochondrial dynamics (fission)	HMSN6B (CMT6B)	AR	Motor or sensory-motor axonal
*MYH14*	Mitochondrial dynamics (fission)	Peripheral neuropathy, myopathy, hoarseness, and hearing loss Deafness, autosomal dominant 4A	AD	Motor axonal (with or without sensory demyelinating changes)
*MFF*	Mitochondrial dynamics (fission)	Encephalopathy due to defective mitochondrial and peroxisomal fission 2	AR	Motor demyelinating or mixed
*DHTKD1*	Mitochondrial energy production (tricarboxylic acid cycle)	CMT2Q	AD	Sensory-motor axonal
*HK1*	Mitochondrial energy production (glycolytic system)	CMT4G	AR	Sensory-motor demyelinating
*COX6A1*	Mitochondrial respiratory chain (complex IV)	CMTRID	AR	Sensory-motor axonal or mixed
*SURF1*	Mitochondrial respiratory chain (complex IV)	CMT4K, Leigh syndrome	AR	Sensory-motor demyelinating
*AIFM1*	Oxidative phosphorylation and redox control in healthy cells	CMTX4 (Cowchock syndrome) Combined oxidative phosphorylation deficiency	XLR	Sensory-motor axonal
*PDK3*	Pyruvate dehydrogenase complex	CMTX6	XLD	Sensory-motor axonal (with or without secondary demyelinating changes)
*C12orf65*	Mitochondrial energy production (oxidative phosphorylation), Mitochondrial translation	Combined oxidative phosphorylation deficiency 7 SPG55, CMT6	AR	Sensory-motor axonal
*POLG1*	mtDNA replication and maintenance	Childhood MCHS, Alpers syndrome ANS disorders, MEMSA, MNGIE-like, SANDO autosomal recessive and dominant PEO	AR and AD	Sensory axonal; hypomyelinating when early onset
*C10orf2* *(Twinkle)*	mtDNA replication and maintenance	ANS disorders	AR and AD	Usually sensory axonal
		Mitochondrial DNA Depletion Syndrome, PEO		
*TYMP*	mtDNA replication and maintenance	Mitochondrial DNA Depletion Syndrome, MNGIE	AR	Sensory-motor demyelinating
*RRM2B*	mtDNA replication and maintenance	Mitochondrial DNA Depletion Syndrome, MNGIE-like, PEO	AR and AD	Sensory-motor demyelinating
*MPV17*	mtDNA maintenance	Mitochondrial DNA Depletion Syndrome Navajo neurohepatopathy	AR	Sensory-motor axonal or demyelinating
*SLC25A19*	mtDNA replication and maintenance	Bilateral striatal degeneration and progressive polyneuropathy	AR	Motor or sensory-motor axonal
*COA7*	Assembling mitochondrial respiratory chain complexes	Spinocerebellar ataxia, autosomal recessive, with axonal neuropathy	AR	Sensory-motor axonal

*AD* autosomal dominant, *ANS* ataxia neuropathy spectrum, *AR* autosomal recessive, *CMT* Charcot–Marie–Tooth disease, *CMTRIA* Charcot–Marie–Tooth disease, recessive intermediate A, *CMTRID* Charcot–Marie–Tooth disease, recessive intermediate D, *HMSN* hereditary motor and sensory neuropathy, *MCHS* myocerebrohepatopathy spectrum disorders, *MEMSA* myoclonus epilepsy myopathy sensory ataxia, *MNGIE* mitochondrial neurogastrointestinal encephalomyopathy, *mtDNA* mitochondrial DNA, *PEO* progressive external ophthalmoplegia, *SANDO* sensory ataxic neuropathy with dysarthria and ophthalmoparesis, *SPAX4* spastic ataxia autosomal recessive Type 4, *SPG55* spastic paraplegia 55, *XLD* X-linked dominant, *XLR* X-linked recessive

In this study of a large Japanese cohort of patients with clinically suspected pure or complex hereditary peripheral neuropathy, we identified novel likely pathogenic/pathogenic variants in four mitochondrial-related nuclear genes. Mitochondrial abnormalities should be considered as a differential diagnosis in cases of axonopathy with suggestive symptoms or other unexplainable multisystem manifestations. Considering the limited number of gene panels targeted in our study, the discovery of more mitochondrial-related nuclear genes leading to mitochondrial-related neuropathy is highly likely. Regarding treatment, early diagnosis would provide more effective and prompt therapy strategies and medicines for the improvement of mitochondrial function might one day be a common target for mitochondrial neuropathy.

## Supplementary Information

Below is the link to the electronic supplementary material.

Supplementary file1 (PDF 156 KB)

## Data Availability

Data are available on request from the authors.
