# Supplementary material for: Complex hereditary peripheral neuropathies caused by novel variants in mitochondrial-related nuclear genes
Source: J Neurol. 2022 Mar 2;269(8):4129–40. doi: 10.1007/s00415-022-11026-w (PMC9293870; doi:10.1007/s00415-022-11026-w)
Supplement: Supplementary file 1 — Supplementary file1 (PDF 156 KB) [file 415_2022_11026_MOESM1_ESM.pdf]

Supplementary Materials

Title: Complex hereditary peripheral neuropathies caused by novel variants in mitochondrial-related nuclear genes

Journal name: Journal of Neurology

Authors:

Yu Hiramatsu, MD,[1] Yuji Okamoto, MD, PhD,[1,2] Akiko Yoshimura, PhD,[1] Jun-Hui Yuan, MD, PhD,[1] Masahiro Ando, MD, PhD[1] Yujiro Higuchi, MD, PhD,[1] Akihiro Hashiguchi, MD, PhD,[1] Eiji Matsuura, MD, PhD,[1] Fumihito Nozaki, MD,[3] Tomohiro Kumada, MD, PhD,[4] Kei Murayama, MD, PhD,[5] Mikiya Suzuki, MD,[6] Yuki Yamamoto, MD,[7] Naoko Matsui, MD, PhD,[7] Yoshimichi Miyazaki, MD,[8] Masamitsu Yamaguchi, PhD,[9] Youji Suzuki, MD,[10] Jun Mitsui, MD, PhD,[11] Hiroyuki Ishiura, MD, PhD,[11] Masaki Tanaka, MD, PhD,[12] Shinichi Morishita, PhD,[13] Ichizo Nishino, MD, PhD,[14] Shoji Tsuji, MD, PhD,[11,12] Hiroshi Takashima, MD, PhD[1]

Affiliations:

[1]Department of Neurology and Geriatrics, Kagoshima University Graduate School of Medical and Dental Sciences, Kagoshima, Japan

[2]Department of Physical Therapy, School of Health Sciences, Faculty of Medicine, Kagoshima University, Kagoshima, Japan

[3]Department of Pediatrics, Shiga Medical Center for Children, Shiga, Japan

[4]Kumada Kids Family Clinic, Shiga, Japan

[5]Department of Metabolism, Chiba Children's Hospital, Chiba, Japan

[6]Department of Neurology, National Hospital Organization Higashisaitama Hospital, Saitama, Japan

[7]Department of Neurology, Tokushima University Graduate School of Medicine, Tokushima, Japan

[8]Department of Neurology, Hyogo Prefectural Awaji Medical Center, Hyogo, Japan

[9]Kansai Gakken Laboratory, Kankyo Eisei Yakuhin Co. Ltd., Seika-cho, Kyoto, Japan

[10]Department of Neurology, Yaizu City Hospital, Shizuoka, Japan

[11]Department of Neurology, Graduate School of Medicine, The University of Tokyo, Tokyo, Japan

[12]Institute of Medical Genomics, International University of Health and Welfare, Chiba, Japan

[13]Department of Computational Biology and Medical Sciences, Graduate School of Frontier Sciences, The University of Tokyo, Chiba, Japan

[14]Department of Neuromuscular Research, National Institute of Neuroscience, National Center of Neurology and Psychiatry (NCNP), Tokyo, Japan

Corresponding author e-mail: thiroshi@m3.kufm.kagoshima-u.ac.jp

Supplementary Table 1: List of 167 screened mitochondria-related nuclear genes.

AARS2	*PHKA1*	*ACAD9*	*DLD*	*MARS2*	*NDUFA1*	*OTC*	*SLC22A5*
ACADL	*POLG1*	*ACAT*	*ETFA*	*MCCC1*	*NDUFA10*	*PC*	*SLC25A13*
ACADM	*POLG2*	*ADCK3*	*ETFB*	*MCCC2*	*NDUFA11*	*PCCA*	*SLC25A15*
ACADVL	*PYGM*	*ARG1*	*ETFDH*	*MGME1*	*NDUFA13*	*PCCB*	*SLC25A19*
AGL	*RARS2*	*ATP5A1*	*ETHE1*	*MMAA*	*NDUFA2*	*PDHA1*	*SLC25A20*
C10orf2	*RRM2B*	*ATP5E*	*FARS2*	*MMAB*	*NDUFA7*	*PDHB*	*SLC25A3*
CPT1B	*SARS2*	*ATPAF2*	*FASTKD2*	*MMACHC*	*NDUFA8*	*PDHX*	*SLC37A4*
CPT2	*SCO2*	*AUH*	*FBXL4*	*MMADHC*	*NDUFAF2*	*PDP1*	*SUCLG2*
DARS2	*SLC25A4*	*BCS1L*	*FOXRED1*	*MPI*	*NDUFAF5*	*PDSS1*	*SURF1*
DGUOK	*SUCLA2*	*BTD*	*G6PC*	*MRPL40*	*NDUFB6*	*PDSS2*	*TACO1*
GAA	*SUCLG1*	*C12orf65*	*GBE1*	*MRPL44*	*NDUFS1*	*PHKA2*	*TCN2*
GYS1	*TAZ*	*COQ2*	*GFM1*	*MRPS16*	*NDUFS2*	*PHKB*	*TFAM*
HADHA	*TK2*	*COQ9*	*GYS2*	*MRPS18A*	*NDUFS3*	*PHKG2*	*TFB1M*
HADHB	*TUFM*	*COX15*	*HLCS*	*MRPS2*	*NDUFS4*	*PMM2*	*TIMM8A*
HARS2	*TYMP*	*COX4I1*	*ISCU*	*MRPS22*	*NDUFS5*	*PUS1*	*TMEM70*
MPV17	*YARS2*	*COX4I2*	*IVD*	*MRRF*	*NDUFS6*	*PYGL*	*TOMM20*
OPA1	*ACADS*	*COX6B1*	*KARS*	*MTFMT*	*NDUFS7*	*SCO1*	*TRMU*
OPA3	*EARS2*	*COX7A1*	*LARS2*	*MTRR*	*NDUFS8*	*SDHAF1*	*TSFM*
PFKM	*MTPAP*	*CPS1*	*LMBRD1*	*MUT*	*NDUFV1*	*SDHAF2*	*UQCRB*
PGAM2	*ACACA*	*CPT1A*	*LPIN1*	*NAGS*	*NDUFV3*	*SDHB*	*UQCRQ*
PGM1	*ACACB*	*DLAT*	*LRPPRC*	*NARS2*	*NUBPL*	*SDHC*	

40 genes colored in white and light gray are myopathy- and rhabdomyolysis-related genes, and 13 genes colored in light gray are also known as mtDNA deletion- or depletion-related genes. In total, 126 genes colored in dark gray are other mitochondria-related nuclear genes, and the *SUCLG2* gene is known as a mtDNA deletion- or depletion-related gene, which leads to encephalomyopathy. mtDNA = mitochondrial DNA.

Supplementary Table 2: Minor allele frequency of validated variants and results of *in silico* analysis identified in this study.

**Patient No.**		**Patient 1**	**Patient 2**		**Patient 3**	**Patient 4**	
Gene symbol		*PDHB*	*MTPAP*		*HADHB*	*SUCLA2*	
Variants		c.880G>A	c.833G>T	c.1531C>T	c.1192T>C	c.1300delG	c.664-1G>A
Amino acid change		p.G294R	p.R278I	p.Q511*	p.F398L	p.D434Mfs*8	p.L222_Q224del
dbSNP155		No	No	No	No	No	No
gnomAD		No	No	No	No	No	No
HGVD		No	No	No	No	No	No
jMorp (8.5K)		No	No	No	No	No	No
In-house database		No	No	No	No	No	No
Segregation		○	○		NA	○	
In silico prediction	PolyPhen-2	0.953	0.980	NA	1.000	NA	NA
	SIFT	0.03	0	NA	0.01	NA	NA
	PROVEAN	−4.173	−7.436	NA	−5.715	NA	−15.779
	Mutation Tester	Disease causing	Disease causing	Disease causing	Disease causing	Disease causing	Disease causing
ACMG	Pathogenicity	PM1, PM2, PP2, PP3, PP4	PM2, PM3, PP1, PP3	PM2, PM4, PP1, PP3	PM1, PM2, PP3, PP4	PVS1, PM2, PM3, PM4, PP3, PP4	PM2, PM3, PM4, PP3, PP4
	Criteria	Likely pathogenic	Likely pathogenic	Likely pathogenic	Likely pathogenic	Pathogenic	Likely pathogenic

The variants in cDNA fragments obtained from RT-PCR of patient 4 are also shown. In-house database comprised whole-exome sequencing and whole-genome sequencing data from 1,253 healthy Japanese

control subjects. In Polymorphism Phenotyping Version 2 (PolyPhen-2), scores >0.5 were considered to be Disease causing. In Sorts Intolerant From Tolerant (SIFT), scores <0.05 are predicted to be deleterious. In Protein Variation Effect Analyzer (PROVEAN), scores <−2.5 are predicted to be deleterious. dbSNP, the single nucleotide polymorphism database (http://www.ncbi.nlm.nih.gov/SNP/); gnomAD browser (April 2021, https://gnomad.broadinstitute.org); HGVD, Human Genetic Variation Database (April 2021, http://www.hgvd.genome.med.kyoto-u.ac.jp); jMorp, Japanese Multi Omics Reference Panel (April 2021, https://jmorp.megabank.tohoku.ac.jp/ijgvd/); NA, not available.
